# Differences in Eye Health, Access to Eye Care Specialists and Use of Lenses among Immigrant and Native-Born Workers in Spain

**DOI:** 10.3390/ijerph16071288

**Published:** 2019-04-10

**Authors:** Mar Seguí-Crespo, Natalia Cantó-Sancho, Alison Reid, José Miguel Martínez, Elena Ronda-Pérez

**Affiliations:** 1 Public Health Research Group, University of Alicante, 03690 San Vicente del Raspeig, Alicante, Spain; mm.segui@ua.es (M.S.-C.); jmartinezma@mc-mutual.com (J.M.M.); elena.ronda@ua.es (E.R.-P.); 2 Department of Optics, Pharmacology and Anatomy, University of Alicante, 03690 San Vicente del Raspeig, Alicante, Spain; 3 School of Public Health, Curtin University, Bentley 6102, Perth, Australia; alison.reid@curtin.edu.au; 4 Department of Statistics, Technical University of Catalonia, 08028 Barcelona, Spain; 5 Area of Healthcare and Economic Benefit, MC Mutual, 08029 Barcelona, Spain; 6 CIBERESP, 28029 Madrid, Spain

**Keywords:** self-perceived eye health, immigrants, Latin Americans, access to eye care specialists, lenses, glasses, Spain

## Abstract

Latin American immigrants make up 49% of the total immigrant population in Spain, yet little is known about their eye health. The aim of this study is to determine if there are differences in self-perceived eye health, access to eye care specialists, and use of lenses between a sample of Latin American immigrant workers from Colombia and Ecuador, and native-born workers in Spain. We used data from the PELFI cohort (Project for Longitudinal Studies of Immigrant Families). The sample consisted of 179 immigrant workers born in Colombia or Ecuador, and 83 Spanish-born workers. The outcome variables were self-perceived eye health, access to eye specialists, and use of lenses. A descriptive analysis of the sample was carried out, and the prevalence of the three outcome variables in immigrants and natives was calculated and adjusted for explanatory variables. Random effects logistic regression models examined eye health outcomes by workers’ country of birth. Immigrants are less likely to report poor self-perceived eye health than native-born (ORc 0.46; CI 95%, 0.22–0.96). Furthermore, they have less access to specialists (ORc 2.61; CI 95%, 1.32–5.15) and a higher probability of needing lenses but not having them (ORc 14.14; CI 95%, 1.77–112.69). This latter variable remained statistically significant after adjusting for covariates (ORa 34.05; CI 95%, 1.59–729.04). Latin American immigrants may not value the use of lenses, despite eye care specialists indicating that they need them. Eye health education is required to recognize the importance of using lenses according to their visual needs.

## 1. Introduction

Eye health is an important facet of general health because of its importance for personal autonomy, carrying out daily activities, and wellbeing. In the work environment, good vision is essential for improving worker productivity and for reducing occupational risk [1,2,3]. Visual impairment, such as uncorrected refractive errors, are associated with a decreased quality of life and pose limitations for activities that depend on seeing, which can lead to reduced educational and work opportunities. Self-perceived health is a good measure for the study of social inequalities in health, as it is a reliable indicator of an individual’s state of health and is strongly associated with the probability of disease risk and use of health services [4,5,6].

Differences in eye health and access to specialist eye care providers based on socioeconomic position has been well studied [7]. However, studies among immigrants are scarce. The few studies that have examined this issue have shown that Latino immigrants in the United States have a high prevalence of visual impairment and undetected eye disease, which is associated with lack of health insurance, failure to visit a specialist, low education level, and lack of acculturation [8]. Similarly, uncorrected refractive errors have also been reported as one of the most prevalent eye health problems among immigrant farm workers in the United States [9]. The percentage of Latino immigrants that have never visited a health specialist to have vision screening ranged from 40% [9] to 75% [10]. Furthermore, just 5% reported the use of lenses (spectacles or contact lenses), although up to 20% reported difficulty seeing in specific situations, such as reading or recognizing a friend on the street [9].

Together with the United States, Spain is the principal destination country (aside from Central America and South America) of Latin American immigrants, who migrate principally for economic and occupational purposes [11,12]. Currently, immigrants make up approximately 10% of Spain’s population. In particular, Latin American immigrants make up 49% of the total immigrant population in Spain [13,14].

Spain recognizes the right to health care for registered immigrants under the same conditions as Spanish citizens [15,16]. Despite this, various studies have shown inequalities in access to care and differences in the use of health services between immigrants and Spaniards [16,17,18,19]. This could be because immigrants’ administrative situation impedes their access to the health system, because they are not aware of existing health services, because they lack economic resources needed to access services (for example, to afford corrective lenses), due to incompatibility of work schedules, or because they may have communication difficulties with health system staff—due to language or cultural differences [20,21].

Moreover, Spain was one of the countries heavily impacted by the 2008 economic crisis, and foreign workers, particularly manual workers, were greatly affected. Economic crises can have negative effects on health through unemployment, job insecurity, and declining household incomes [22]. Although there are no specific references about the potential additional adverse effects of a financial crisis on visual health, access to eye care specialists, and use of lenses, other studies from Spain (conducted during this time period) highlighted an increase in barriers to health service utilization by migrants [23] and a deterioration in their living and working conditions and health [24].

For the reasons listed above, the objectives of this study are to determine whether there are differences in self-perceived eye health, access to eye care specialists, and use of lenses (spectacles or contact lenses) in a sample of Latin American working immigrants from Colombia and Ecuador, compared with native-born workers in Spain.

## 2. Methods

### 2.1. Design and Study Population

The data for this study came from the PELFI cohort (Project for Longitudinal Studies of Immigrant Families). PELFI is a multi-center project that evaluates the effect of the migratory process on different determinants of health. PELFI is made up of a prospective cohort of 250 families (193 immigrant families, and 57 native-born families) in two Spanish cities (Alicante and Barcelona). The cohort is based on a convenience sample recruited through key informants [25]. Recruitment took place in June 2015. To date there have been two follow-up waves, in 2016 and 2017. Very briefly, with respect to the reference sample of PELFI, 250 families were recruited—82 from Ecuador, 82 from Colombia, 29 from Morocco, and 57 from Spain. A total of 283 women and 190 men were interviewed. All adults had a health card, 67.1% of them had Spanish nationality, and 26.8% had permanent residence or a work permit (or it was being processed). They had an average of 13 years residence in Spain at the time of the interview (± 4.38 years), although more than 50% had been living in the country for more than 14 years.

The definition of family was defined using that of the Spanish National Health Survey (ENSE, 2011/2012) [26]: Persons who occupy a household—or a part of one—with shared consumption and budget (they share food or other items purchased with a common budget) and who have resided together for at least six months at the time of recruitment. Immigrant families were considered to be those in which both parents (father or mother for single-parent families) were born in Colombia or Ecuador. The selection criteria for a family included having at least one son/daughter aged between 12 and 17 years, and at least one adult aged 18 to 65 years who had worked for at least one year in Spain, not necessarily continuously. Families had to expect to reside in Spain for at least 18 months after being selected for the study. Families were only included in the sample when all of their members who complied with the selection criteria were interviewed. All families (immigrants and native-born) were recruited from neighborhoods with high levels of foreigners and low levels of economic resources, compared with the total for the respective municipalities. Within each family, all of the adults were interviewed in their homes, or at associations or public places in the neighborhood, by professional, trained interviewers. The interviewers had experience working with immigrant populations, and, when possible, with immigrants from the same country of origin as the families. The questionnaire was designed ad hoc, with questions about sociodemographic and family characteristics, the migratory process, social support, working and living conditions, and mental health. The questions about eye health were added during the second follow-up.

The project was approved by the ethics committee of the University of Alicante (UA-2014-06-26). All procedures performed in this study were in accordance with the 1964 Helsinki declaration and its later amendments or comparable ethical standards. Data were anonymized, ensuring confidentiality required by Spanish law on the Protection of Personal Data.

For this analysis, only those actively working at the time of the interview were included. The sample was made up of a total of 262 workers, of whom 179 were Latin American and 83 were native-born.

### 2.2. Variables Related to Eye Health

Variables related to eye health were the following: (a) Self-perceived eye health. Each worker reported what he/she considered to be his/her state of eye health or vision in general, with five response options (1—very poor; 2—poor; 3—regular; 4—good; 5—very good), which were regrouped into poor (1, 2, and 3) and good (4 and 5) eye health. (b) Access to eye care specialists. Each worker indicated the date of his/her last visit to an eye care specialist (an ophthalmologist or optician-optometrist) for a check-up, advice, or treatment for problems related to eye health or vision, with four response options (1—never; 2—less than one year prior; 3—between one and three years prior; 4—more than three years prior). In Spain, there are two pathways to access eye care specialists, one leads to an ophthalmologist and the other to an optician. Both are free of charge. (c) Use of lenses (spectacles/contact lenses). Each participant reported whether a specialist had ever recommended he/she use spectacles or contact lenses, with five response options: (1—yes, but he/she had undergone eye surgery and no longer needed them; 2—yes, he/she has them and wears them regularly; 3—yes, he/she has them but doesn’t often wear them; 4—yes, but he/she doesn’t have them; 5—the specialist indicated he/she doesn’t need them) where only the affirmative responses (1, 2, 3, and 4) were used as variable categories. The variable unmet need for lenses corresponds to affirmative category 4.

The following were included as explanatory variables: a) Sociodemographic variables—age (18–40, 41–46, >46 years), sex (male, female), and education level (none, primary, secondary, or university education). b) Occupational variables—occupational social class was based on the participant’s current job title [27] (i.e., the occupation (job) at the time of the interview). It was coded as per the Spanish National Classification of Occupations-2011 with the following categories: Management workers, technicians and professionals, support technicians and professionals, office workers, services and sales workers, qualified agriculture and fishing workers, industrial qualified workers, operators and assemblers, and unqualified workers. For the analysis, this variable was recoded into non-manual and manual occupations. Data were also gathered on the type of work day (normal, irregular), whether the worker’s salary covers important unforeseen expenses (no, yes), whether the job allows him/her to leave for a doctor’s visit when needed (no, yes), and net monthly salary (≤451 €, 452–751 €, 752–1503 €, >1503 €).

For all of the variables there was the option to respond don’t know/no answer (DK/NA).

### 2.3. Statistical Analysis

A descriptive analysis was carried out of all sociodemographic and occupational factors by worker origin (immigrants born in Colombia or Ecuador and those born in Spain). Prevalence of self-perceived eye health was calculated, as well as access to eye care specialists and use of lenses, using the worker’s origin as the explanatory variable, and sociodemographic and occupational variables. Statistically significant differences in the obtained prevalences were examined using Fisher’s exact test. Finally, for each health result (poor self-perceived eye health, limited access to a specialist >3 years or never, and the unmet need for corrective lenses), by worker’s country of origin, logistic regression models were carried out with family-specific random effects. The association measure obtained was odds ratios (OR) with confidence intervals of 95%. Both crude OR (ORc) and adjusted OR (ORa) by all of the explanatory variables and the study city, were calculated. The programs SPSS version 15 (IBM, Armonk, NY, USA) and Stata version 10 (College Station, TX, USA) were used.

## 3. Results

There is a greater proportion of women in the study than men (immigrants, 67% and Spaniards, 53%) (Table 1). A greater proportion of Spanish had a university education (37.3%) compared with immigrants (17.3%). The majority of workers had regular workdays. Almost half of the immigrant workers indicated that their salary did now allow them to cover unforeseen expenses.

In contrast, 81.7% of native-born workers had a salary that would cover unforeseen expenses, and they also were allowed to leave work to visit the doctor. Immigrants’ salaries were lower than native’s salaries (Table 1).

The prevalence of self-perceived good eye health was greater in immigrants than in Spaniards (62% compared with 47%) (Table 2). However, there was a high percentage of immigrants who had never visited an eye care specialist (15.2%) or whose last visit was more than three years prior to the interview (20.8%). For those born in Spain, for whom the prevalence of poor self-perceived vision health was 53%, the majority had visited a specialist less than one year prior or between one and three years prior to the interview (81.9%). A greater percentage of those born in Spain who reported needing lenses have them, and wear spectacles or contact lenses regularly (71.4%) compared with 32.7% of immigrants. There were also differences between groups in the unmet need for lenses—1.3% of the native-born reported unmet need compared with 15.3% of immigrants (Table 2).

Native-born men reported a greater prevalence of poor self-perceived eye health (48%) compared with immigrant workers (27.1%) (Table 3). In both groups, there was a greater prevalence of poor self-perceived eye health for those who reported insufficient salaries to cover unforeseen expenses. Statistically significant differences were found in the group that reported that they could leave their work to visit the doctor, and among this group there was greater prevalence of poor self-perceived eye health in the Spanish-born (51.3%) compared with immigrants (37.4%). The greatest prevalence of poor self-perceived eye health was found among the native-born, among the group that earned a monthly net salary of 452–751 euros.

Among those who had not visited a specialist for more than three years or never, female immigrants had the least access to specialists. Similarly, the youngest workers (18–40 years) had the least access to a specialist, with 48% of immigrants having not seen a specialist for three years or more or never, compared with (25.0%) of those born in Spain for this age group. There was a greater prevalence in reduced access to a specialist among workers who responded that they could not leave work to visit a doctor. However, statistically significant differences were found among the group that reported being able to leave work in order to visit a doctor. There was a greater prevalence of reduced access to a specialist in immigrants (35.1%) compared with those born in Spain (17.1%).

Men in our study were more likely than women to need lenses and not have them, this occurred more in immigrants than in those born in Spain. There were statistically significant differences among the group aged 41–46 years, for whom no one born in Spain reported an unmet need for lenses, compared with 20.5% of immigrants who reported an unmet need.

When workers’ salaries were sufficient to cover unforeseen expenses, there was a greater prevalence of unmet need for lenses among immigrants, and this was even greater in those that reported insufficient salaries for covering unforeseen expenses (21.1%). Among those who could leave work to visit the doctor, there was a greater unmet need for lenses among immigrants than those born in Spain, 13.7% and 1.4%, respectively (Table 3). 

Immigrants had a lower probability of reporting poor self-perceived eye health than the native-born (ORc 0.46; CI 95%, 0.22–0.96; *p*-value, 0.039) (Table 4). However, this group had the least access to a specialist (ORc 2.61; CI 95%, 1.32–5.15; *p*-value, 0.006) and a greater probability of unmet need for lenses (ORc 14.14; CI 95%, 1.77–112.69; *p*-value, 0.012) compared with those born in Spain. After adjusting for sociodemographic and occupational variables, unmet need for lenses is the only variable that remains statistically significant and different between both groups (ORa 34.05; CI 95%, 1.59–729.04; *p*-value, 0.024) (Table 4).

## 4. Discussion

This study found a similar prevalence of poor self-perceived eye health and access to an eye care specialist among immigrants and Spanish-born workers. The unmet need for lenses was more frequent among immigrant workers than among native-born workers.

Other studies conducted in the United States, among a sample of more than 6000 self-identified Latinos of 40 years of age and older, have shown that Latinos have a high prevalence of visual impairment, uncorrected refractive errors, and unmet need for lenses, and conclude that these problems could decrease as a result of greater education and access to eye care specialists among the Latino population [28,29]. These studies, like many studies conducted in the United States, examine differences in the use of health services by ethnicity or race, but do not examine differences by immigration status. Immigrant’s reported the best self-perceived eye health in our study, which differs from another study carried out in Spain [17], in which the immigrant population showed worse self-perceived health than natives. These differences can be explained by the duration of residence in the host country. In our case, the immigrant population was settled in Spain, where more than 50% had been living in the country for more than 14 years. While in the other study, 65.2% of the Latin American immigrants had been residing in Spain for less than 3 years. Our findings concur with the results of a multi-center study among primary health care centers in Spain, which showed that settled Latin American immigrants aged between 18 and 55 reported better health-related quality of life than did the Spanish-born. The authors suggested that sharing a language (Spanish) and cultural similarities could act as facilitating factors in the migratory process [30]. However, the differences found with the studies carried out in the United States could—on the one hand—be due to the fact that in the United States, Latino immigrants don’t speak English as a native language. On the other hand, the differences may be due to the fact that in our study we only included those actively working, which could reflect a selection bias known as the ‘healthy immigrant effect’—immigrants with better health than natives [31,32]—which has been observed for many years in many countries [33,34].

There were statistically significant differences in access to eye care specialists between groups, but these disappeared after the models were adjusted by sociodemographic and occupational variables. Other studies in Spain have signaled that immigrants from different parts of the world (United States, Central and South America, Africa, Asia, among others) use specialist services without public assistance (e.g. dentists) less frequently than the Spanish population [35]. The authors suggest several different reasons: First, the administrative burden for accessing specialist services whose complexity limits immigrants’ use of services. Second, cultural reasons—it is possible that immigrants retain the same pattern of behavior with regard to the health services use in the host country as in their country of origin. In this regard, the lack of universal health coverage in the countries of immigrants from Latin America may explain the lower use of health services among immigrants. Lastly, as has been mentioned, long working days resulting from precarious work contracts can also be a barrier to accessing these services.

The reduced access to specialist services (ophthalmologists/opticians) that is observed in our results by Latin Americans may be explained because one of the factors that influences access to eye care services is need [9,36,37]. More than 50% of immigrants in this study reported having good self-perceived eye health, and they might therefore consider that they do not have any vision problems or worrisome symptoms, and therefore there is no need to seek out a specialist. This was observed in another study, where 42.1% of immigrant farmworkers who reported not having seen a vision specialist in the past year indicated that they did not need it and had no reason to go [9].

Regarding the use of lenses, there were differences between those born in Spain and those born in Colombia or Ecuador. Only 32.7% of Latin Americans reported using lenses and wearing them frequently compared to 71.4% of the native-born. Furthermore, 15.3% of immigrants had an unmet need for lenses. Differences in cultural integration, levels of education, or economic activities could explain this. Immigrants earned lower salaries than those born in Spain (approximately 65% earned less than 751 euros per month compared with 23.4% of the Spanish-born), almost 50% indicated that their salary wasn’t sufficient to cover unforeseen expenses (compared with 18.3% of the native-born), and they had lower levels of education (17.3% have a university education, compared with 37.3% of the native-born). Another cross-sectional study, in which 1235 Hispanic/Latino participants were surveyed, found that the Hispanic/Latino population with higher education were more likely to have knowledge about and access to information about eye health [38], which would probably encourage them to acquire spectacles or contact lenses to compensate their failing vision. However, in the current study, these differences remained after adjusting for sociodemographic and occupational variables. Above all, the high probability of immigrants to have an unmet need for lenses compared with the native-born signals that country of birth (being an immigrant or not) should be considered an important factor, independent of other social, economic, or occupational determinants.

The results of this study should be interpreted with some caution due to some study limitations. Although PELFI is a longitudinal study, the current study of eye health has a transversal design, which limits the ability to establish a causal relationship between variables. The information about access to eye care specialists could be subject to recall bias, and the estimated self-perceived state of eye health could be inaccurate, given that it wasn’t measured objectively [10,39]. However, self-perceived health is used frequently in epidemiological studies because of its validity in predicting morbidity and mortality and its use in following population groups with specific health problems, in addition to the measurement of intervention effectiveness [40]. We modelled those whose last visit to an eye care specialist was three years or more prior to the interview date or never, with the aim of studying the most unfavorable group. In general, it is not easy to establish the ideal time frame for visiting an eye health specialist. The National College of Opticians/Optometrists (CNOO) indicates that such visits should occur annually. The American Academy of Ophthalmology (AAO) recommends that high-risk populations, including the Latino population, carry out an eye exam every one to three years from age 40 to 54, which is the age group of the majority of our study population [41]. We also need to consider that although the sampling occurred across areas of the city, we cannot rule out some degree of selection bias due to the non-probabilistic sampling techniques. However, the sampling techniques applied in this study have been suggested as the most useful for recruiting immigrant populations into cohort studies [42]. In addition, PELFI is a cohort study which contains participants who are in settled families and who have lived in Spain for an average of 13 years. Therefore, our results are not representative of the most vulnerable groups of migrants (e.g., newcomers and illegal immigrants). Furthermore, the results from this current study would not be representative of those immigrants from North Africa and other countries, who do not speak Spanish as their native language. In the current study we studied immigrants from Colombia and Ecuador, which may have resulted in the sample lacking representativeness of the general immigrant population in Spain and could affect the external validity of the results. Despite the limitations, the principal strength of this study lies in the fact that it provides a first examination of the determinants of eye health among workers born in Colombia or Ecuador, and those born in Spain. To date, studies carried out in Spain have been focused on evaluating inequalities in general health between immigrants and the native-born rather than the determinants of those inequalities.

## 5. Conclusions

In this study, immigrants were found to have fewer economic resources than Spaniards. Importantly, this lack of resources may be an influential barrier that stops them acquiring lenses. However, differences in the acquisition of lenses remain between migrant and native-born after adjusting for sociodemographic and occupational variables, which suggests that other determinants may also influence the unmet need for corrective lenses. Those born in Colombia or Ecuador might not value the use of lenses needed to correct refractive error, even when a vision health specialist recommends it.

In conclusion, our results suggest that Latin American Immigrants report better self-perceived eye health and lower access to eye care specialists than Spaniards. Yet, country of origin (being a Latin American immigrant or not) does not explain these differences. In contrast, country of origin influences the need for corrective lenses. Immigrants had a greater risk of unmet need for lenses to correct refractive defects.

Therefore, given the importance of visual health for workers, visual health education is required—especially aimed at this group of immigrants—to reduce inequality in visual health and emphasize the importance of attending vision specialists to perform periodic reviews. In addition, future studies are required to evaluate what other determinants could be influencing the inequalities found in our results.

## Figures and Tables

**Table 1:** Distribution of workers included in the study by sociodemographic characteristics, occupational characteristics, and country of origin.

Variables	Total		Born in Spain		Born in Colombia or Ecuador		*p*-Value ^A^
	*n*	(%)	*n*	(%)	*n*	(%)	
Sex							
Female	164	(62.6)	44	(53.0)	120	(67.0)	0.039 *
Male	98	(37.4)	39	(47.0)	59	(33.0)	
Age							
18–40 years	85	(32.4)	12	(14.5)	73	(40.8)	<0.001 ***
41–46 years	78	(29.8)	27	(32.5)	51	(28.5)	
>46 years	99	(37.8)	44	(53.0)	55	(30.7)	
Education level							
University education	62	(23.7)	31	(37.3)	31	(17.3)	0.002 **
Secondary education	151	(57.6)	38	(45.8)	113	(63.1)	
None or primary education	49	(18.7)	14	(16.9)	35	(19.6)	
Occupational social class							
Non-manual	61	(23.1)	49	(58.5)	12	(6.7)	<0.001 ***
Manual	201	(76.9)	34	(41.5)	167	(93.3)	
Type of work day ^B^							
Regular	167	(64.0)	63	(75.9)	104	(58.4)	0.008 **
Irregular	94	(36.0)	20	(24.1)	74	(41.6)	
Salary unforeseen expenses ^B^							
No	96	(38.4)	15	(18.3)	81	(48.2)	<0.001 ***
Yes	154	(61.6)	67	(81.7)	87	(51.8)	
Work permits doctor visits							
No	24	(9.4)	4	(5.0)	20	(11.4)	0.112
Yes	231	(90.6)	76	(95.0)	155	(88.6)	
Net monthly salary ^B^							
≤451 Euros	63	(24.7)	15	(18.5)	48	(27.6)	<0.001 ***
452–751 Euros	68	(26.7)	4	(4.9)	64	(36.8)	
752–1503 Euros	91	(35.7)	34	(42.0)	57	(32.8)	
>1503 Euros	33	(12.9)	28	(34.6)	5	(2.9)	
Total	262	(100.0)	83	(100.0)	179	(100.0)	

^A^ Fisher’s exact test. ^B^ Variables with missing values. Note: * *p* < 0.05, ** *p* < 0.01, *** *p* < 0.001.

**Table 2:** Prevalence of self-perceived eye health, access to a specialist (ophthalmologist/optician-optometrist), and use of lenses (spectacles/contact lenses) in workers included in the study by sociodemographic and occupational characteristics.

Variables	Total		Born in Spain		Born in Colombia or Ecuador		*p*-Value ^A^
	*n*	(%)	*n*	(%)	*n*	(%)	
Self-perceived eye health							
Good	150	(57.3)	39	(47.0)	111	(62.0)	0.031 *
Poor	112	(42.7)	44	(53.0)	68	(38.0)	
Access to specialist ^B^							
Never	32	(12.3)	5	(6.0)	27	(15.2)	0.026 *
Less than one year prior	105	(40.2)	41	(49.4)	64	(36.0)	
From 1 to 3 years prior	77	(29.5)	27	(32.5)	50	(28.1)	
More than 3 years prior	47	(18.0)	10	(12.0)	37	(20.8)	
Use of lenses ^C^ (spectacles/contact lenses)							
Yes, but does not need them	52	(22.9)	10	(13.0)	42	(28.0)	<0.001 **
Yes, and wears them regularly	104	(45.8)	55	(71.4)	49	(32.7)	
Yes, has them but does not wear them	47	(20.7)	11	(14.3)	36	(24.0)	
Yes, but does not have them	24	(10.6)	1	(1.3)	23	(15.3)	

^A^ Fisher’s exact test. ^B^ Variable with a missing value. ^C^ 35 Workers in the sample were told by a specialist that they did not need corrective lenses or Don’t know/Refused. Note: * *p* < 0.05, ** *p* < 0.001.

**Table 3:** Prevalence of poor self-perceived eye health, access to an eye care specialist (ophthalmologist/optician–optometrist) more than three years prior or never, and unmet need for lenses in workers included in the study by sociodemographic and occupational variables and country of origin.

Variables		Poor Eye Health							Access to a Specialist More Than 3 Years Prior or Never							Unmet Need for Spectacles/Contact Lenses						
		Total		Born in Spain		Born in Colombia or Ecuador		*p*-Value ^A^	Total		Born in Spain		Born in Colombia or Ecuador		*p*-Value ^A^	Total		Born in Spain		Born in Colombia or Ecuador		*p*-Value ^A^
		*n*	%	*n*	%	*n*	%		*n*	%	n	%	*n*	%		*n*	%	*n*	%	*n*	%	
Sex																						
	Female	77	47.0	25	56.8	52	43.3	0.158	48	29.3	7	15.9	41	34.2	0.032 *	14	9.7	0	0.0	14	13.7	0.011 *
	Male	35	35.7	19	48.7	16	27.1	0.034 *	31	32.0	8	20.5	23	39.7	0.075	10	12.0	1	2.9	9	18.8	0.039 *
Age																						
	18–40 years	21	24.7	3	25.0	18	24.7	1.000	35	41.2	3	25.0	32	43.8	0.344	7	10.9	0	0.0	7	13.2	0.339
	41–46 years	37	47.4	15	55.6	22	43.1	0.346	21	26.9	6	22.2	15	29.4	0.597	9	13.4	0	0.0	9	20.5	0.023 *
	>46 years	54	54.5	26	59.1	28	50.9	0.543	23	23.5	6	13.6	17	31.5	0.055	8	8.3	1	2.3	7	13.2	0.071
Education level																						
	University	35	56.5	18	58.1	17	54.8	1.000	6	9.7	2	6.5	4	12.9	0.671	4	6.5	0	0.0	4	12.9	0.113
	Secondary	55	36.4	17	44.7	38	33.6	0.245	57	38.0	9	23.7	48	42.9	0.052	12	9.8	1	2.9	11	12.5	0.176
	No education or primary education	22	44.9	9	64.3	13	37.1	0.116	16	32.7	4	28.6	12	34.3	1.000	8	19.0	0	0.0	8	25.8	0.086
Occupational social class																						
	Non-Manual	31	51.7	27	56.3	4	33.3	0.204	12	11.7	5	10.4	7	58.3	0.001 **	1	1.8	0	0.0	1	11.1	0.164
	Manual	80	40.0	17	50.0	63	38.0	0.249	66	33.2	10	29.4	56	33.9	0.692	23	13.5	1	3.3	22	15.6	0.083
Type of work day																						
	Regular	74	44.3	33	52.4	41	39.4	0.111	42	25.3	8	12.7	34	33.0	0.003 **	17	11.1	0	0.0	17	18.5	<0.001 ***
	Irregular	37	39.4	11	55.0	26	35.1	0.127	36	38.3	7	35.0	29	39.2	0.800	7	9.5	1	6.3	6	10.3	1.000
Salary for unforeseen expenses																						
	No	46	47.9	11	73.3	35	43.2	0.048 *	31	32.3	3	20.0	28	34.6	0.372	15	17.4	0	0.0	15	21.1	0.063
	Yes	61	39.6	33	49.3	28	32.3	0.046 *	43	28.1	12	17.9	31	36.0	0.018 *	8	6.1	1	1.6	7	9.9	0.068
Work permits doctor visit																						
	No	11	45.8	3	75.0	8	40.0	0.300	10	41.7	2	50.0	8	40.0	1.000	5	26.3	0	0.0	5	31.3	0.530
	Yes	97	42.0	39	51.3	58	37.4	0.048 *	67	29.1	13	17.1	54	35.1	0.005 **	19	9.4	1	1.4	18	13.7	0.004 **
Net monthly salary																						
	≤451 Euros	27	42.9	8	53.3	19	39.6	0.384	23	36.5	5	33.3	18	37.5	1.000	5	9.8	0	0.0	5	13.2	0.311
	452–751 Euros	33	48.5	3	75.0	30	46.9	0.349	22	32.8	0	0.0	22	34.9	0.294	11	19.3	0	0.0	11	20.8	0.577
	752–1503 Euros	30	33.0	18	52.9	12	21.1	0.003 **	28	30.8	7	20.6	21	36.8	0.158	5	6.3	0	0.0	5	10.0	0.151
	>1503 Euros	19	57.6	15	53.6	4	80.0	0.336	3	9.1	2	7.1	1	20.0	0.400	2	6.1	1	3.6	1	20.0	0.284
Total		112	42.7	44	53.0	68	38.0	0.031 *	79	30.3	15	18.1	64	36.0	0.004 **	24	57.1	1	50.0	23	57.5	<0.001 ***

^A^ Fisher’s exact test. Note: * *p* < 0.05, ** *p* < 0.01, *** *p* < 0.001.

**Table 4:** Logistic regression model of poor eye health, access to a specialist more than three years prior or never, and unmet need for corrective lenses in workers included in the study by country of origin.

		ORc ^A^	(CI 95%)	*p*-Value	ORa ^B^	(CI 95%)	*p*-Value
Poor eye health	Born in Spain ^C^	1			1		
	Born in Colombia or Ecuador	0.46	(0.22–1.96)	0.039 *	0.52	(0.21–1.32)	0.170
Access to a specialist more than three years prior or never	Born in Spain ^C^	1			1		
	Born in Colombia or Ecuador	2.61	(1.32–5.15)	0.006 **	1.73	(0.72–4.18)	0.223
Has unmet need for glasses/contact lenses	Born in Spain ^C^	1			1		
	Born in Colombia or Ecuador	14.14	(1.77–112.69)	0.012 *	34.05	(1.59–729.04)	0.024 *

^A^ Crude Odds Ratio. ^B^ Adjusted Odds Ratio by sociodemographic and occupational variable. ^C^ Reference group; * *p* < 0.05, ** *p* < 0.01.
